# Effects of Prenatal Breastfeeding Education on Breastfeeding Duration Beyond 12 Weeks: A Systematic Review

**DOI:** 10.1177/10901981231220668

**Published:** 2024-01-19

**Authors:** Megan K. Oggero, Cathy L. Rozmus, Geri LoBiondo-Wood

**Affiliations:** 1 Cizik School of Nursing, The University of Texas Health Science Center at Houston, Houston, TX, USA; 2 McGovern Medical School, The University of Texas Health Science Center at Houston, Houston, TX, USA

**Keywords:** breastfeeding, breastfeeding duration, breastfeeding education, healthy people programs, prenatal, psychology

## Abstract

The proportion of infants in the United States who are breastfed at 1 year remains well below the Healthy People 2030 target. The health implications of suboptimal breastfeeding durations are significant, including increased risk of childhood leukemia and maternal Type 2 diabetes. Prenatal breastfeeding education provides an opportunity to improve breastfeeding self-efficacy among pregnant individuals and to establish their coping skills in case future breastfeeding problems arise. Although prenatal breastfeeding education is known to improve breastfeeding self-efficacy, characteristics of prenatal breastfeeding education interventions that are successful at increasing breastfeeding duration have not been well defined. Using Preferred Reporting Items for Systematic Reviews and Meta-Analyses guidelines and the Health Action Process Approach, we conducted a systematic review of the literature examining the impact of prenatal breastfeeding education interventions on breastfeeding duration measured at least 12 weeks postpartum. Twenty-one studies were identified. Prenatal breastfeeding education was most likely to increase breastfeeding duration when education interventions integrated psychological components (Health Action Process Approach coping planning) or were paired with in-person postpartum breastfeeding support. Additional research is needed to examine the role of psychological components in breastfeeding education interventions in diverse populations and to determine the specific psychological intervention components with the greatest impact on breastfeeding duration.

Breastfeeding is promoted as the ideal source of infant nutrition by health organizations worldwide (Centers for Disease Control and Prevention, 2021; United Nations International Children’s Emergency Fund [UNICEF], 2018; World Health Organization [WHO], 2022). Breastfeeding confers numerous health benefits for both parents and infants, often in a dose-response manner (Meek et al., 2022; Qiao et al., 2020; Unar-Munguía et al., 2017). Examples include a 23% lower risk of childhood leukemia among infants with the longest breastfeeding durations and a 9% reduction in relative risk of Type 2 diabetes for mothers for each 12-month increase in lifetime breastfeeding duration (Aune et al., 2014; Su et al., 2021). Breastfeeding duration is the length of time an infant receives any breast milk (Lumbiganon et al., 2016). Various time points are used to assess duration (e.g., 6 and 12 weeks, Abbass-Dick et al., 2015; 3 and 6 months, Efrat et al., 2015). A breastfeeding duration of at least 2 years is encouraged (Meek et al., 2022; WHO, 2021).

Although breastfeeding initiation rates in the United States have remained above 80% for the past decade, the proportion of infants breastfed at 1 year is currently estimated at 35.9%, well below the Healthy People 2030 target of 54.1% (National Center for Chronic Disease Prevention and Health Promotion (NCCDPHP), 2022; Office of Disease Prevention and Health Promotion (ODPHP), 2020). Many prenatal breastfeeding education interventions have been designed to improve breastfeeding duration, but the outcomes have varied widely (Wong et al., 2015). Characteristics of breastfeeding education interventions that are successful at affecting breastfeeding duration have not been well defined. However, prenatal breastfeeding education is important, as it helps prepare expecting parents for breastfeeding and improves breastfeeding self-efficacy (Brockway et al., 2017).

Prenatal breastfeeding education occurs during pregnancy in various formats (Lumbiganon et al., 2016; Wong et al., 2015). It is often measured categorically based on group assignment using a dichotomous or multi-group categorization (Antoñanzas-Baztán et al., 2021; Kellams et al., 2018). Survival analysis is commonly used to measure breastfeeding duration.

Previous research on the relationship between prenatal breastfeeding education and breastfeeding duration was examined in a Cochrane review (Lumbiganon et al., 2016). The authors determined that there was no conclusive evidence supporting the role of prenatal breastfeeding education in improving breastfeeding duration. However, they excluded studies whose interventions occurred partially outside of the prenatal period, and 13 of the 20 included studies had breastfeeding education or support as part of the standard care comparator. Wong et al. (2021), in a systematic review and meta-analysis of the effectiveness of educational and supportive interventions on breastfeeding outcomes among primiparous women, concluded that educational and supportive interventions were effective at increasing breastfeeding duration up to 2 months postpartum but not 3–5 months or ≥6 months postpartum. However, in 8 of the 13 included studies, the interventions were conducted exclusively in the postpartum period. In addition, the authors included only studies in which participants gave birth vaginally. In 2021, approximately one third of births in the United States occurred via cesarean section (National Center for Health Statistics, 2023). Limiting studies by birth type limits the generalizability of their findings, as there are significant differences between women who deliver vaginally and women who give birth by cesarean section (Witt et al., 2015).

Before additional funding is allocated for breastfeeding education intervention development, a thorough analysis of the existing literature examining the effect of prenatal breastfeeding education on breastfeeding duration is needed. The purpose of this review was to synthesize the published research on prenatal breastfeeding education and its effect on breastfeeding duration. Therefore, the question addressed in this systematic review was, “In pregnant women, do breastfeeding education interventions have an effect on breastfeeding duration?”

## Theoretical Framework

**Figure 1.**
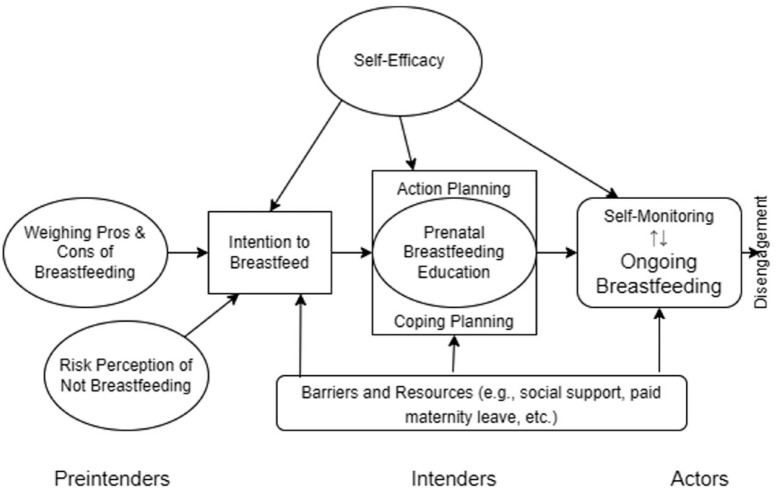
Health Action Process Approach to Prenatal Breastfeeding Education and Breastfeeding Duration.

The Health Action Process Approach (HAPA) is a theory of health behavior change that aims to explain, predict, and modify health behaviors (Schwarzer, 2016). HAPA integrates action planning and coping planning as mediators between intention and behavior, providing targets for interventions that may affect breastfeeding duration (Figure 1). A key HAPA principle is that intentions are more likely to become behaviors when detailed plans are created, success scenarios are visualized, and preparatory strategies for conquering challenges are developed. Action plans address the when, where, and how, and coping plans serve a compensatory function by representing alternatives to the action plan. In breastfeeding education, action planning occurs through basic education on topics such as positioning and latch. However, considering the high incidence of breastfeeding problems experienced by lactating individuals, the coping planning portion of a breastfeeding education intervention is expected to have the largest impact on breastfeeding outcomes (Gianni et al., 2019). Coping planning could involve identifying potential breastfeeding problems, providing customized plans to address them, and equipping individuals with psychological skills that facilitate perseverance.

In this review, HAPA was the framework by which breastfeeding education interventions were evaluated. Because individuals commonly encounter problems at some point in the course of breastfeeding, we expected that participants in studies whose breastfeeding education interventions incorporated coping planning elements would have longer breastfeeding durations than participants in studies whose interventions did not (Gianni et al., 2019). In addition, studies incorporating postnatal support would likely reflect longer breastfeeding durations owing to the role of the postnatal support (as action control and social support) in shielding the goal-pursuit process from derailing interference (Schwarzer, 2016).

## Method

The literature search plan for this systematic review was developed in consultation with an academic research librarian (Lefebvre et al., 2019). The Preferred Reporting Items for Systematic Reviews and Meta-Analyses (PRISMA) guidelines directed the planning and reporting of this review (Page, McKenzie, et al., 2021; Page, Moher, et al., 2021). The primary unit of analysis in this review was studies rather than individual reports (Higgins et al., 2019).

The databases searched were PubMed, MEDLINE, Embase, the Cumulative Index to Nursing and Allied Health Literature, and the Cochrane Library. Because technological and cultural shifts occur over time and reduce the applicability of past research to the present population, this review was limited to articles published in the past 15 years (January 1, 2008, through January 30, 2023; Charlesworth & Banaji, 2019).

Four topic groups and their key terms were used to search the databases. The topic groups were (a) antenatal, antepartum, or prenatal; (b) education, intervention, support, or teaching; (c) breast feeding, breastfeeding, or infant feeding; and (d) duration or time. First, the Medical Subject Headings and key terms in each topic group were combined in an “OR” statement. Each major topic group was also combined with an “AND” statement. The reference lists of selected studies and related systematic reviews were reviewed to identify additional publications.

Studies were included if they (a) included pregnant women as participants, (b) used a prenatal breastfeeding education intervention with or without intrapartum or postpartum components, (c) had a comparison group that did not receive the primary education intervention, (d) reported rates of breastfeeding duration at 12 weeks or greater, (e) were a randomized controlled trial (RCT) or quasi-experimental study, and (f) were published in English. Studies were excluded if expectant fathers, grandparents, or non-maternal individuals were the only target audience for the intervention. Studies were not excluded based on the type, frequency, or duration of the breastfeeding education interventions, provided at least a portion occurred prenatally. Because the acceptability of breastfeeding in individuals with HIV varies worldwide, studies specifically targeting HIV-positive individuals were excluded (Bansaccal et al., 2020).

**Figure 2.**
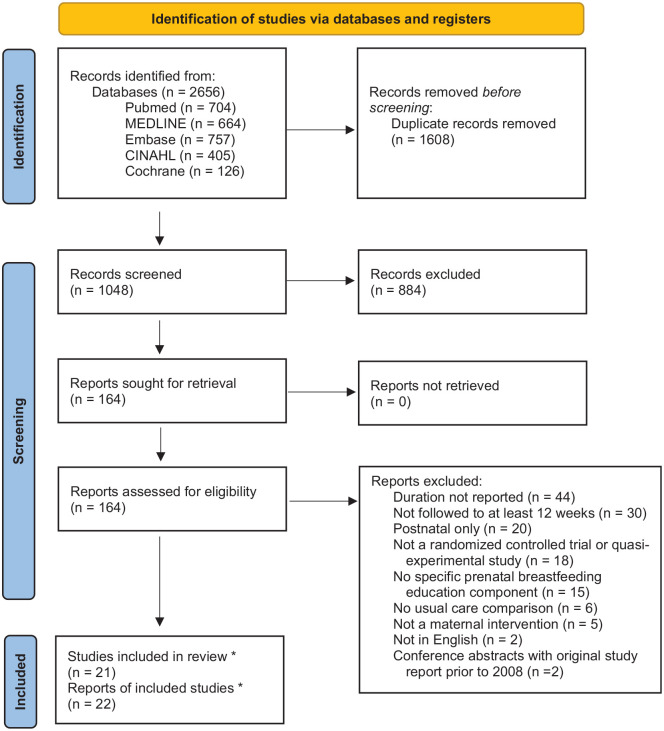
PRISMA Flow Diagram. *Note.* Twenty-two reports meeting the inclusion criteria were included. However, two reports *were* published about the same study findings; one was a full report and the other a conference abstract. Only the full report was evaluated in this review, resulting in 21 included studies. PRISMA = Preferred Reporting Items for Systematic Reviews And Meta-Analyses; CINAHL = Cumulative Index to Nursing and Allied Health Literature.

A PRISMA flow diagram outlining the study screening process is presented in Figure 2 (Page, McKenzie, et al., 2021). In the initial screening, the titles and abstracts of all identified studies (*n* = 1,048) were reviewed against all inclusion and exclusion criteria. Studies were excluded if any violations were found. Full-text articles of the remaining studies (*n* = 164) were further evaluated against the inclusion and exclusion criteria, and additional studies (*n* = 142) were excluded. Ultimately, 21 studies were included.

### Data Extraction and Synthesis

Information was extracted for narrative synthesis using a form designed by the first author. For each study, the form consolidated information on study design, purpose, setting, sample, interventions, usual care or control characteristics, efforts to ensure intervention fidelity, measurement of breastfeeding duration, data quality, statistical tests, findings, author recommendations, and strengths and weaknesses. The completed forms and publications were iteratively evaluated to identify themes.

### Study Quality Assessment

Quality scores for RCTs were calculated using the van Tulder scale for RCTs (van Tulder et al., 2003). Studies were considered of sufficient quality if the total score was 5 or higher. Quality scores for quasi-experimental studies were calculated using the Joanna Briggs Institute (JBI, 2020) Critical Appraisal Checklist for Quasi-Experimental Studies. Studies were considered of sufficient quality if the total score was 7 or higher (Remington & Winters, 2019).

## Results

### Characteristics of the Studies

This review included 21 studies; of those, 16 studies were RCTs, and 5 studies used quasi-experimental designs (see Table 1 for full details). Sample sizes ranged from 45 to 1,193, with a median sample size of 362. The studies were conducted in 10 different countries and used convenience sampling.

**Table 1.** Study Characteristics.

Study	Study type	Location	Sample size	Participant age (years)		Socioeconomic status	Specific population (if any)
				*M*	*SD*		
Antoñanzas-Baztán et al. (2021)	QE	Spain	123	34.5	6.2	Mid to high	
Bonuck et al. (2014)	RCT	United States	628	27.9	5.9	Low	
Cauble et al. (2021)	RCT	United States	45	26.0	4.3	Mid to high	
Chan et al. (2016)	RCT	Hong Kong	71	32.0	3.9	Mid to high	
Efrat et al. (2015)	RCT	United States	289	27.5	6.0	Low	Low-income, Hispanic women
Jiang et al. (2014)	QE	China	582	59.5% 25–29^a^		Mid to high	Nulliparous women
Kellams et al. (2018)	RCT	United States	522	25.2	5.8	Low	
Kronborg et al. (2012)	RCT	Denmark	1,193	29.0	3.7	Mid to high	
Meedya et al. (2014)	QE	Australia	366	26.6	4.9	Mixed	Nulliparous women
Mikami et al. (2017)	RCT	Brazil	171	28.3	6.0	Mixed	Women pregnant with twins
Nabulsi et al. (2019)	RCT	Lebanon	362	29.6	4.9	Mid to high	
Puharić et al. (2020)	RCT	Croatia	400	71% 25–35^a^		Mixed	Nulliparous women
Schreck et al. (2017)	QE	United States	650	25.8	10.7	Low	
Stuebe et al. (2016)	RCT	United States	100	30.2	6.3	Mixed	Women with GDM
Tseng et al. (2020)	RCT	Taiwan	104	33.1	4.2	Mid to high	
van Dellen et al. (2019)	QE	The Netherlands	138	31.5	4.4	Mid to high	
Wambach et al. (2011)	RCT	United States	390	17.0	0.9	Low	Pregnant adolescents
Wen et al. (2011)	RCT	Australia	667	26.0	5.5	Mixed	
Wen et al. (2020)	RCT	Australia	1,155	32.5	5.0	Mid to high	
Wong et al. (2014)	RCT	Hong Kong	469	31.4	4.3	Mixed	Nulliparous women
Zhao et al. (2021)	RCT	China	182	30.5	3.3	Mid to high	Women with depressive symptoms

*Note. M* = mean; *SD* = standard deviation; QE = quasi-experimental; RCT = randomized controlled trial; GDM = gestational diabetes mellitus.

a Mean and standard deviation not reported; values shown are the percentage of participants in the largest age category.

The socioeconomic status (SES) of participants varied across studies. Although SES was not measured identically in all studies, sufficient metrics were reported for broad categorization. Five studies had samples from low-income populations, all conducted in the United States. In most cases, participants were considered to have low incomes if enrolled in Medicaid or the Special Supplemental Nutrition Program for Women, Infants, and Children (WIC). Ten studies had participants with characteristics indicating moderate to high SES. In these studies, education level was a proxy for SES. Studies with moderate to high SES samples had >50% of participants with a history of university attendance or a bachelor’s degree (see Supplemental Table S3 for additional details). In two studies, specific educational attainment was unreported, but the authors reported that participants had a high education level. In the six remaining studies, participants were socioeconomically diverse, reporting a mixed distribution of education or income.

Seven studies had prenatal interventions only, and 13 had interventions with components in both the prenatal and postpartum periods (see Table 2 for full details). One additional study evaluated participants who completed only the prenatal portion of the intervention and separately evaluated participants who completed the prenatal portion and attended an optional postpartum breastfeeding support group (Schreck et al., 2017). In the studies with prenatal interventions only, interventions either integrated psychological interventions with breastfeeding education content or focused exclusively on breastfeeding education content. The studies with both prenatal and postpartum intervention components combined prenatal breastfeeding education content with postnatal support and additional education. Intervention details are found in Table 3 and in the Supplemental Material.

**Table 2.** Components of Study Interventions.

Study	Prenatal interventions								Postpartum interventions				Significant effect on breastfeeding duration^a^
	Handouts or booklet	Video	In-person individual education	In-person group education	Individual phone education	Group phone education	Text messages	Psychological (coping planning)	In-person individual support	In-person group support	Phone support	Text messages	
Antoñanzas-Baztán et al. (2021)	x	x							x		x		Yes
Bonuck et al. (2014)			x						x		x		Yes
Cauble et al. (2021) ^b^	x					x							No
Chan et al. (2016)				x				x			x		No
Efrat et al. (2015)					x						x		No
Jiang et al. (2014)							x					x	No
Kellams et al. (2018) ^b^		x											No
Kronborg et al. (2012) ^b^				x									No
Meedya et al. (2014)	x	x		x				x			x		Yes
Mikami et al. (2017) ^b^				x									No
Nabulsi et al. (2019)	x	x		x					x		x		No
Puharić et al. (2020)	x				x			x			x		Yes
Schreck et al. (2017)	x		x							x			Yes
Stuebe et al. (2016)				x			x			x		x	Yes
Tseng et al. (2020) ^b^				x				x					Yes
van Dellen et al. (2019)			x						x		x		Yes
Wambach et al. (2011)				x	x				x		x		Yes
Wen et al. (2011)			x						x				Yes
Wen et al. (2020) ^c^	x				x		x				x		No
Wong et al. (2014) ^b^	x		x										No
Zhao et al. (2021) ^b^			x					x					Yes

a Study intervention was considered to have a significant effect on breastfeeding duration if *p* ≤ .05 or if the confidence interval did not contain 1. ^b^Studies with prenatal interventions only. ^c^Intervention group also received a mailed booklet to review prior to phone call.

**Table 3.** Description of Study Interventions.

Study	Intervention description	Control description
Antoñanzas-Baztán et al. (2021)	Participants given written information and viewed BSE-enhancing video during weeks 28–39 of pregnancy; during hospitalization after birth, given specific advice on items with a low score on BSES-SF and observation of whole BFG; within 48–72 hours after discharge, follow-up call with verbal advice on low BSES-SF items	Routine antenatal and PP visits
Bonuck et al. (2014)	*EP*: EPs appear in EMR during 5 prenatal visits to prompt discussion by HCP *LC*: Two prenatal sessions, hospital visit, regular phone calls PP through 3 months or until BFG ceased, PP home visits optional *LC+EP*: both EP and LC interventions	Access to routine hospital IBCLC
Cauble et al. (2021)	Six 60-min sessions of prenatal group phone counseling with 6–10 participants led by IBCLC, assigned tasks for subsequent week	Standard pregnancy and pediatric education by HCP
Chan et al. (2016)	2.5-hr interactive BFG workshop at 28–38 weeks GA, groups of 6–8; Telephone counseling at 2 weeks PP, each call 30–60 min long	BFG support from midwives in the hospital, PP follow-up
Efrat et al. (2015)	Four prenatal and 17 PP phone calls (most 5–7 min), 2 prenatal contacts focused on equipping with critical BFG knowledge (about 20 min)	Routine BFG education and support offered by health system
Jiang et al. (2014)	Once weekly text message with BFG or infant feeding advice from 28 weeks GA to 12 months PP	Usual healthcare services
Kellams et al. (2018)	25-min educational BFG video viewed at prenatal visit	20-min educational nutrition/exercise video
Kronborg et al. (2012)	Ready for Child program; 3 modules, 3 hr each at 30–35 weeks GA, partner invited, up to 8 couples per class, lecture and discussions; BFG portion about 2 hr long	Standard care offered by the antenatal clinic
Meedya et al. (2014)	Milky Way program elements: group sessions, take-home learning activities, and postnatal telephone consultations to support BFG (one within the first 10 days and the other about 3 months PP). Group sessions started in the second trimester. Women and partner/support person participated in groups of 10–20 people.	Standard maternity care
Mikami et al. (2017)	Three 30-min small group BFG-specific counseling sessions of 2–3 women provided by 1 of 2 midwives	Routine antenatal care protocol for twin pregnancies
Nabulsi et al. (2019)	Three components; (a) prenatal BFG education, (b) PP professional lactation support, (c) PP peer (lay) support	Standard prenatal and postnatal care provided by obstetricians only
Puharić et al. (2020)	Received BFG booklet and general pregnancy booklet, followed by 4 proactive phone calls—1 in pregnancy and 3 PP at 2, 6, and 10 weeks	*Active control*: received general pregnancy booklet, followed by 4 proactive phone calls—1 in pregnancy and 3 PP at 2, 6, and 10 weeks *Usual care*: received standard care
Schreck et al. (2017)	BFG-focused prenatal education curriculum of up to 10 visits at site-associated resident prenatal clinic delivered one-on-one by an IBCLC; subgroup optionally attended BFG support group	Care in prenatal clinic before intervention implementation
Stuebe et al. (2016)	NEST group intervention beginning with a prenatal BFG class. Then, starting about 6 weeks PP, women participated in a 13-week intensive lifestyle intervention delivered as weekly classes and a home exercise program.	Usual care for lactation support and gestational diabetes
Tseng et al. (2020)	Integrated BFG education program based on theory of self-efficacy, three 2.5-hr sessions of 4–5 couples at 34, 35, and 36 weeks GA, simulation and mindfulness components	Standard usual care provided at study site hospital based on Baby Friendly Hospital Initiative
van Dellen et al. (2019)	Series of 6 consults up to 10 weeks PP, mix of in-person and phone calls	Usual obstetric and PP care
Wambach et al. (2011)	Prenatal, in-hospital, and PP education and support. 2 prenatal classes (one 1.5-hr, one 2 hr), peer counselor calls before and after class 1 and after class 2. Visit from peer counselor in hospital after birth; BFG teens visited by IBCLC; PP phone contact at 4, 7, 11, 18 days, and 4 weeks	*Attention control*: paralleled the intervention group in content amount/timing; no BFG focus *Usual care*: received standard prenatal and PP care at their respective clinic
Wen et al. (2011)	One home visit at 30–36 weeks GA and 5 home visits at 1, 3, 5, 9, and 12 months PP. At each visit, research nurse spent 1–2 hours with the dyad	Usual childhood nursing service, 1 home visit within a month of birth if needed
Wen et al. (2020)	*Both intervention groups*: 1 intervention in third trimester and 5 interventions at 1, 3, 5, 7, and 10 months PP. Staged booklets mailed to match timing of support *Phone intervention*: 1 week post-booklet, phone call (30–60 min) to support, discuss booklet, address issues *SMS intervention*: 1 week after booklet, messages sent twice weekly for 4 weeks via 2-way SMS to reinforce key messages	Usual care from local health nurses, home safety materials sent to control group in third trimester, 3, 6, and 9 months PP
Wong et al. (2014)	20- to 30-min 1-to-1 BFG education and support session based on World Health Organization guidelines; delivered immediately after randomization in a private room in the antenatal clinics, handouts given	Standard antenatal care
Zhao et al. (2021)	Individualized mixed management psychoeducational intervention focused on perinatal mental health and BFG, delivered in four 60-min face-to-face sessions	Routine obstetric examination and follow-up

*Note.* BSE = breastfeeding self-efficacy; BSES-SF = Breastfeeding Self-Efficacy Scale—Short Form; BFG = breastfeeding; PP = postpartum; EP = electronic prompts; EMR = electronic medical record; HCP = health care provider; IBCLC = International Board-Certified Lactation Consultant; LC = lactation consultant; GA = gestational age; NEST = nutrition, exercise, and social cognitive theory-based.

### Findings

In 11 of the 21 included studies, interventions showed significant positive effects on breastfeeding duration measured at 12 or more weeks postpartum (see Table 2). Iterative evaluation of the included studies led to the identification of two major themes: intervention timing and intervention type. Intervention timing reflects whether the intervention was specific to the prenatal period or had components in both the prenatal and postpartum periods. Intervention type is evaluated in the context of intervention timing.

#### Intervention Timing

Of the 11 studies whose interventions had significant positive effects on breastfeeding duration, 9 had interventions with a combination of prenatal and postpartum components, and 2 had interventions with only prenatal components. Of the 14 studies whose interventions had both prenatal and postpartum components, 9 (64%) had significant effects on breastfeeding duration. However, significant effects on breastfeeding duration were observed in only two (29%) of the seven studies whose interventions had only prenatal components.

#### Intervention Type

##### Studies With Prenatal Intervention Components Only

Among the seven studies with prenatal interventions only, the two with significant positive effects on breastfeeding duration integrated psychological interventions into their breastfeeding education interventions. Couples-based breastfeeding education with mindfulness training and simulation exercises demonstrated a significant positive effect on breastfeeding duration to 3 months postpartum with an effect approaching significance at 6 months postpartum (Tseng et al., 2020). The prenatal intervention with the most sustained effect on breastfeeding duration significantly affected breastfeeding duration to 6 months postpartum using a combined psychological education and simulation intervention (Zhao et al., 2021). Both studies were conducted in East Asian countries with moderate to high SES samples and included participants who gave birth vaginally or by cesarean section. No effect on breastfeeding duration was identified among studies whose prenatal-only interventions did not integrate psychological practices.

##### Studies With Both Prenatal and Postpartum Intervention Components

Among the 14 studies with prenatal and postpartum intervention components, 9 studies had interventions with significant positive effects on breastfeeding duration and 7 of those used in-person support in the postpartum period. The two studies that did not use in-person postpartum support but whose interventions significantly affected breastfeeding duration used prenatal interventions that integrated psychological components with postpartum phone support. One study integrated an interactive activity in which participants received education about relaxation using music, breathing techniques, and visualization during three 90-min antenatal breastfeeding education sessions (Meedya et al., 2014, 2016). Another study integrated psychological components of behavior change, such as goal setting, commitment, confidence boosting, and establishing social support, into a single antenatal phone call (Puharić et al., 2020; Zakarija-Grković et al., 2017). Both studies included participants of mixed SES and who gave birth vaginally or by cesarean section.

One additional study with general breastfeeding education and psychological components (education about coping and reinforcement of coping strategies) with postpartum phone support showed an effect on breastfeeding duration that approached significance (Chan et al., 2016). Although almost twice as many mothers in the intervention group were breastfeeding their infants at 6 months compared with those in the control group, the study’s small sample size resulted in low power and an insignificant *p* value despite a clinically relevant difference between the groups.

Only one study with an in-person postpartum support component did not identify a significant effect on breastfeeding duration (Nabulsi et al., 2019). However, only 22.4% of intervention group participants completed all three intervention components, and only 30% completed the prenatal education component.

### Quality Assessment

Among the RCTs, van Tulder scores ranged from 4 to 10. Fourteen RCTs were of sufficient quality, as indicated by van Tulder scores of 5 or higher. JBI scores ranged from 7 to 8. All five quasi-experimental studies were of sufficient quality, as indicated by JBI scores of 7 or higher. All 11 studies whose interventions positively affected breastfeeding duration were of sufficient quality. Only 2 of the 21 included studies were low quality, and neither had interventions with a significant positive effect on breastfeeding duration (Efrat et al., 2015; Mikami et al., 2017).

## Discussion

Based on this review, two primary conclusions can be made regarding the effect of prenatal breastfeeding education interventions on breastfeeding duration. First, prenatal breastfeeding education alone has the potential to impact breastfeeding duration but appears effective only when psychological components are integrated. Second, prenatal breastfeeding education interventions are more likely to be effective when paired with in-person postnatal support. These findings are consistent with HAPA concepts (Schwarzer, 2016). Breastfeeding duration was longer among the participants in breastfeeding education interventions with psychological elements than among participants in studies with breastfeeding education alone. Psychological components may help participants develop coping skills that facilitate perseverance when encountering breastfeeding difficulties.

Combined prenatal and postpartum breastfeeding interventions appear more effective when postpartum breastfeeding support occurs in-person instead of via telephone or text messaging. Postpartum support components serve as built-in coping planning elements with the potential to assist if breastfeeding problems arise. HAPA does not explain why in-person support is more effective than telephone or text message support. However, breastfeeding is a hands-on activity; logically, in-person support may have a more significant effect than other forms, owing to the opportunity for hands-on assistance. In addition, no studies have demonstrated the equivalence of telephone, text, or telehealth support compared with in-person support regarding breastfeeding duration outcomes (Gavine et al., 2022).

Although a Cochrane review (Lumbiganon et al., 2016) evaluating the relationship between prenatal breastfeeding education and breastfeeding duration determined that there was no conclusive evidence supporting the role of prenatal breastfeeding education in improving breastfeeding duration, only one of the included studies had a prenatal breastfeeding education intervention with a psychological component (Noel-Weiss, Rupp, et al., 2006). That single study’s intervention included discussion of coping in the postpartum period by emphasizing the need to allow time for healing, recovery, and role adjustment, but it does not appear that the researchers related coping techniques to breastfeeding or breastfeeding challenges (Noel-Weiss, Bassett, & Cragg, 2006). The study findings were somewhat inconclusive as there was no difference in breastfeeding duration measured at 8 weeks postpartum using intent-to-treat analysis, but a statistically significant and clinically relevant difference was found in the per-protocol analysis (Noel-Weiss, Rupp, et al., 2006). Because the authors give insufficient detail to determine the nature and extent of the coping discussion or its potential impact on breastfeeding duration, the findings of this single study neither support nor contradict the potential utility of psychological interventions in improving breastfeeding duration.

Given the lack of studies with psychological intervention components in the Cochrane review, it is unsurprising that the authors of the Cochrane review found no effect of prenatal breastfeeding education on breastfeeding duration. Because this current review (a) included studies published more recently than those included in the Cochrane review, (b) did not exclude studies with postpartum intervention components, and (c) was guided by HAPA, we were able to identify a trend toward significantly increased breastfeeding duration when prenatal breastfeeding education interventions included psychological components. Similarly, by including studies with women of all parities and delivery types, this current review evaluated a broader range of prenatal breastfeeding education interventions than Wong et al. (2021), who limited their review to include only studies with primiparous women who delivered vaginally and concluded that breastfeeding education interventions were effective at increasing breastfeeding duration only up to 2 months postpartum.

The integration of psychological components as coping planning into prenatal breastfeeding education interventions shows promise in any context but may be especially helpful in populations with poor access to in-person postpartum lactation support. For patients in rural settings, there is often a lack of local lactation support. Integrating psychological components into a prenatal breastfeeding education curriculum that is available remotely may help partially compensate for the lack of available in-person support by strengthening coping skills preemptively. Pairing prenatal breastfeeding education using psychological components that promote coping with postpartum breastfeeding support delivered by phone or through a video-based telehealth system may help to extend breastfeeding duration among rural populations where in-person breastfeeding assistance is unavailable.

Coping planning is not exclusive to the prenatal period. Therefore, physicians, nurses, and other providers, particularly those in obstetric and pediatric ambulatory clinics and antepartum, postpartum, and neonatal hospital units, are ideally situated to assist with forms of coping planning that may increase breastfeeding success rates and lengthen breastfeeding duration among their patients. Similarly, public health organizations and perinatal family support organizations such as the Nurse–Family Partnership and WIC are uniquely situated to use coping planning principles to support breastfeeding across the perinatal period.

### Strengths and Weaknesses

The research examining the effects of breastfeeding education interventions on breastfeeding duration has several strengths. The majority of the studies had large samples, with 12 enrolling over 350 participants each. In addition, most studies used intent-to-treat analyses. Many of the studies compared breastfeeding duration between intervention and control groups using survival analysis. Survival analysis allows greater statistical power to be achieved while accounting for censored observations, which are ignored in many other statistical tests (George et al., 2014). Finally, most of the studies monitored participants for at least 6 months, and several monitored them for a year. Monitoring participants for longer durations provides a more precise picture of an intervention’s overall effect on breastfeeding duration.

High rate of participants who were lost to follow-up in several studies is a significant weakness of the research evaluating the effects of breastfeeding education interventions on breastfeeding duration. In seven studies, more than 20% of participants were lost to follow-up, which poses a serious threat to validity (Sackett et al., 2000). In one study, 37% of intervention group participants and 47% of control group participants were lost to follow-up by the 6-month data collection point (Antoñanzas-Baztán et al., 2021), and in another study, 52% of intervention group participants and 46% of control group participants were lost to follow-up by the 10-month data collection point (Stuebe et al., 2016). In addition, most studies had restrictive inclusion and exclusion criteria, resulting in samples that were not representative of the general population of pregnant women; therefore, the generalizability of their findings was limited. About half of the studies restricted inclusion to low-risk pregnant women with a singleton gestation and no complicating or potentially complicating conditions. All studies relied on participant self-report of breastfeeding duration. While measuring breastfeeding duration via participant self-report is typical, it may be biased. Because participants, particularly those in the intervention groups, were exposed to messages that positively promoted breastfeeding, responses may have been influenced by social desirability bias (Cresswell et al., 2019). In addition, breastfeeding duration is often reported weeks to months after breastfeeding cessation, and participants may have been prone to recall bias (Abdel-Hady & El-Gilany, 2016).

All studies with significant positive findings involved time-intensive and costly interventions. Although finding potentially effective interventions is promising, most of these interventions are unrealistic for implementation in a non-research setting. Interventions that are time-intensive for staff and patients may not be well accepted or easily implemented in the general population, may not be scalable, and may lack sufficient sustainability (Klaic et al., 2022). Finally, with the increasing use and desirability of virtual education and health care services, the absence of studies using virtual meeting environments or providing breastfeeding support via video-based telehealth is a significant limitation of the current literature.

### Implications for Research

The current body of literature evaluating the impact of prenatal breastfeeding education interventions on breastfeeding duration highlights a need for research examining (a) the role of psychological components in breastfeeding education interventions, (b) the effectiveness of combining prenatal breastfeeding education interventions with psychological components and in-person postpartum support, and (c) the effectiveness of prenatal education and postpartum support components delivered via video-based telehealth. The two included studies with significant positive effects on breastfeeding duration whose interventions were exclusive to the prenatal period and included psychological components were conducted in East Asian countries with relatively small samples (Tseng et al., 2020; Zhao et al., 2021). Future research is needed to evaluate prenatal breastfeeding education interventions with psychological components in more diverse populations and to determine the specific psychological intervention components with the greatest impact on breastfeeding outcomes. In addition, no studies paired prenatal breastfeeding education with a psychological component and postpartum in-person breastfeeding support. These interventions appear effective independently and may be especially effective when combined. Future research should examine these interventions for a possible synergistic effect.

All studies whose interventions had significant positive effects on breastfeeding duration used interventions that were time-intensive for both participants and research staff. Additional research is needed to determine if similar but shorter interventions might have similar effectiveness. Developing similar but more feasible interventions is critical to facilitate sustainability (Klaic et al., 2022).

Finally, none of the studies used online meeting platforms or other forms of web- or app-based delivery to implement their prenatal breastfeeding education intervention, and none used video-based telehealth to provide postpartum breastfeeding support. The feasibility, acceptability, and effectiveness of these technologies should also be explored in the context of prenatal breastfeeding education, postpartum breastfeeding support, and breastfeeding duration outcomes.

## Conclusion

Prenatal breastfeeding education is most likely to increase breastfeeding duration compared with usual care when psychological components are integrated or it is paired with in-person postpartum support. However, these interventions are time-intensive, potentially costly, and may not be feasible on a large scale as currently designed. Additional research is needed to develop similar interventions that can be implemented on a large scale without compromising effectiveness. This review offers a new contribution by integrating HAPA action planning and coping planning concepts into the context of prenatal breastfeeding education through combined educational and psychological intervention components.

## Supplemental Material

sj-docx-1-heb-10.1177_10901981231220668 – Supplemental material for Effects of Prenatal Breastfeeding Education on Breastfeeding Duration Beyond 12 Weeks: A Systematic Review Supplemental material, sj-docx-1-heb-10.1177_10901981231220668 for Effects of Prenatal Breastfeeding Education on Breastfeeding Duration Beyond 12 Weeks: A Systematic Review by Megan K. Oggero, Cathy L. Rozmus and Geri LoBiondo-Wood in Health Education & Behavior
