# Supplementary material for: Effects of Prenatal Breastfeeding Education on Breastfeeding Duration Beyond 12 Weeks: A Systematic Review
Source: Health Educ Behav. 2024 Jan 19;51(5):665–76. doi: 10.1177/10901981231220668 (PMC11420594; doi:10.1177/10901981231220668)
Supplement: sj-docx-1-heb-10.1177_10901981231220668 – Supplemental material for Effects of Prenatal Breastfeeding Education on Breastfeeding Duration Beyond 12 Weeks: A Systematic Review [file sj-docx-1-heb-10.1177_10901981231220668.docx]

**Supplementary Files**

**Table S1**

*Overview of Breastfeeding Benefits for Infants and Parents*

| Benefits for breastfeeding infants | References |
| --- | --- |
| Reduced risks of: |  |
| Asthma | Dogaru, C. M., Nyffenegger, D., Pescatore, A. M., Spycher, B. D., & Kuehni, C. E. (2014). Breastfeeding and childhood asthma: Systematic review and meta-analysis. *American Journal of Epidemiology*, *179*(10), 1153–1167. <https://doi.org/10.1093/aje/kwu072>  Lodge, C. J., Tan, D. J., Lau, M. X. Z., Dai, X., Tham, R., Lowe, A. J., Bowatte, G., Allen, K. J., & Dharmage, S. C. (2015). Breastfeeding and asthma and allergies: A systematic review and meta-analysis. *Acta Paediatrica*, *104*(467), 38–53. <https://doi.org/10.1111/apa.13132> |
| Obesity | Horta, B. L., Loret de Mola, C., & Victora, C. G. (2015). Long-term consequences of breastfeeding on cholesterol, obesity, systolic blood pressure and type 2 diabetes: A systematic review and meta-analysis. *Acta Paediatrica*, *104*(467), 30–37. <https://doi.org/10.1111/apa.13133>  Rito, A. I., Buoncristiano, M., Spinelli, A., Salanave, B., Kunešová, M., Hejgaard, T., García Solano, M., Fijałkowska, A., Sturua, L., Hyska, J., Kelleher, C., Duleva, V., Musić Milanović, S., Farrugia Sant’Angelo, V., Abdrakhmanova, S., Kujundzic, E., Peterkova, V., Gualtieri, A., Pudule, I., … Breda, J. (2019). Association between characteristics at birth, breastfeeding and obesity in 22 countries: The WHO European Childhood Obesity Surveillance Initiative - COSI 2015/2017. *Obesity Facts*, *12*(2), 226–243. <https://doi.org/10.1159/000500425> |
| Type 1 diabetes | Al Mamun, A., O’Callaghan, M. J., Williams, G. M., Najman, J. M., Callaway, L., & McIntyre, H. D. (2015). Breastfeeding is protective to diabetes risk in young adults: A longitudinal study. *Acta Diabetologica*, *52*(5), 837–844. <https://doi.org/10.1007/s00592-014-0690-z>  Lund-Blix, N. A., Dydensborg Sander, S., Størdal, K., Nybo Andersen, A.-M., Rønningen, K. S., Joner, G., Skrivarhaug, T., Njølstad, P. R., Husby, S., & Stene, L. C. (2017). Infant feeding and risk of type 1 diabetes in two large Scandinavian birth cohorts. *Diabetes Care*, *40*(7), 920–927. <https://doi.org/10.2337/dc17-0016> |
| Type 2 diabetes | Horta, B. L., & de Lima, N. P. (2019). Breastfeeding and type 2 diabetes: Systematic review and meta-analysis. *Current Diabetes Reports*, *19*(1), 1. <https://doi.org/10.1007/s11892-019-1121-x> |
| Sudden infant death syndrome | Thompson, J. M. D., Tanabe, K., Moon, R. Y., Mitchell, E. A., McGarvey, C., Tappin, D., Blair, P. S., & Hauck, F. R. (2017). Duration of breastfeeding and risk of SIDS: An individual participant data meta-analysis. *Pediatrics*, *140*(5), e20171324. <https://doi.org/10.1542/peds.2017-1324> |
| Improved cognitive development | Cai, S., Pang, W. W., Low, Y. L., Sim, L. W., Sam, S. C., Bruntraeger, M. B., Wong, E. Q., Fok, D., Broekman, B. F. P., Singh, L., Richmond, J., Agarwal, P., Qiu, A., Saw, S. M., Yap, F., Godfrey, K. M., Gluckman, P. D., Chong, Y.-S., Meaney, M. J., … GUSTO Study Group. (2015). Infant feeding effects on early neurocognitive development in Asian children. *The American Journal of Clinical Nutrition*, *101*(2), 326–336. <https://doi.org/10.3945/ajcn.114.095414>  Horta, B. L., Loret de Mola, C., & Victora, C. G. (2015). Breastfeeding and intelligence: A systematic review and meta-analysis. *Acta Paediatrica*, *104*(467), 14–19. <https://doi.org/10.1111/apa.13139>  Victora, C. G., Bahl, R., Barros, A. J. D., França, G. V. A., Horton, S., Krasevec, J., Murch, S., Sankar, M. J., Walker, N., & Rollins, N. C. (2016). Breastfeeding in the 21st century: Epidemiology, mechanisms, and lifelong effect. *The Lancet*, *387*(10017), 475–490. <https://doi.org/10/cv86> |
| Decreased incidence of respiratory tract infections and diarrheal illnesses | Quigley, M. A., Carson, C., Sacker, A., & Kelly, Y. (2016). Exclusive breastfeeding duration and infant infection. *European Journal of Clinical Nutrition*, *70*(12), 1420–1427. <https://doi.org/10.1038/ejcn.2016.135>  Raheem, R. A., Binns, C. W., & Chih, H. J. (2017). Protective effects of breastfeeding against acute respiratory tract infections and diarrhoea: Findings of a cohort study. *Journal of Paediatrics and Child Health*, *53*(3), 271–276. <https://doi.org/10/f92xr6> |
| Overall decrease in rates of neonatal and infant mortality | Li, R., Ware, J., Chen, A., Nelson, J. M., Kmet, J. M., Parks, S. E., Morrow, A. L., Chen, J., & Perrine, C. G. (2022). Breastfeeding and post-perinatal infant deaths in the United States, A national prospective cohort analysis. *The Lancet Regional Health – Americas*, *5*. <https://doi.org/10.1016/j.lana.2021.100094>  Sankar, M. J., Sinha, B., Chowdhury, R., Bhandari, N., Taneja, S., Martines, J., & Bahl, R. (2015). Optimal breastfeeding practices and infant and child mortality: A systematic review and meta-analysis. *Acta Paediatrica*, *104*(467), 3–13. <https://doi.org/10.1111/apa.13147> |
| Benefits for breastfeeding parents | References |
| Reduced risks of: |  |
| High blood pressure | Qu, G., Wang, L., Tang, X., Wu, W., & Sun, Y. (2018). Association between duration of breastfeeding and maternal hypertension: A systematic review and meta-analysis. *Breastfeeding Medicine*, *13*(5), 318–326. <https://doi.org/10.1089/bfm.2017.0180>  Rameez, R. M., Sadana, D., Kaur, S., Ahmed, T., Patel, J., Khan, M. S., Misbah, S., Simonson, M. T., Riaz, H., & Ahmed, H. M. (2019). Association of maternal lactation with diabetes and hypertension: A systematic review and meta-analysis. *JAMA Network Open*, *2*(10), e1913401. <https://doi.org/10.1001/jamanetworkopen.2019.13401> |
| Type 2 diabetes | Aune, D., Norat, T., Romundstad, P., & Vatten, L. J. (2014). Breastfeeding and the maternal risk of type 2 diabetes: A systematic review and dose–response meta-analysis of cohort studies. *Nutrition, Metabolism and Cardiovascular Diseases*, *24*(2), 107–115. <https://doi.org/10.1016/j.numecd.2013.10.028>  Chowdhury, R., Sinha, B., Sankar, M. J., Taneja, S., Bhandari, N., Rollins, N., Bahl, R., & Martines, J. (2015). Breastfeeding and maternal health outcomes: A systematic review and meta-analysis. *Acta Paediatrica*, *104*(467), 96–113. <https://doi.org/10.1111/apa.13102>  Jäger, S., Jacobs, S., Kröger, J., Fritsche, A., Schienkiewitz, A., Rubin, D., Boeing, H., & Schulze, M. B. (2014). Breast-feeding and maternal risk of type 2 diabetes: A prospective study and meta-analysis. *Diabetologia*, *57*(7), 1355–1365. <https://doi.org/10.1007/s00125-014-3247-3>  Rameez, R. M., Sadana, D., Kaur, S., Ahmed, T., Patel, J., Khan, M. S., Misbah, S., Simonson, M. T., Riaz, H., & Ahmed, H. M. (2019). Association of maternal lactation with diabetes and hypertension: A systematic review and meta-analysis. *JAMA Network Open*, *2*(10), e1913401. <https://doi.org/10.1001/jamanetworkopen.2019.13401> |
| Breast and ovarian cancers | Babic, A., Sasamoto, N., Rosner, B. A., Tworoger, S. S., Jordan, S. J., Risch, H. A., Harris, H. R., Rossing, M. A., Doherty, J. A., Fortner, R. T., Chang-Claude, J., Goodman, M. T., Thompson, P. J., Moysich, K. B., Ness, R. B., Kjaer, S. K., Jensen, A., Schildkraut, J. M., Titus, L. J., … Terry, K. L. (2020). Association between breastfeeding and ovarian cancer risk. *JAMA Oncology*, *6*(6), e200421. <https://doi.org/10/drcv>  Unar-Munguía, M., Torres-Mejía, G., Colchero, M. A., & González de Cosío, T. (2017). Breastfeeding mode and risk of breast cancer: A dose–response meta-analysis. *Journal of Human Lactation*, *33*(2), 422–434. <https://doi.org/10.1177/0890334416683676> |
| Obesity | Sharma, A. J., Dee, D. L., & Harden, S. M. (2014). Adherence to breastfeeding guidelines and maternal weight 6 years after delivery. *Pediatrics*, *134*(0 1), S42–S49. <https://doi.org/10.1542/peds.2014-0646H>  Wiklund, P., Xu, L., Lyytikäinen, A., Saltevo, J., Wang, Q., Völgyi, E., Munukka, E., Cheng, S., Alen, M., Keinänen-Kiukaanniemi, S., & Cheng, S. (2012). Prolonged breast-feeding protects mothers from later-life obesity and related cardio-metabolic disorders. *Public Health Nutrition*, *15*(1), 67–74. <https://doi.org/10.1017/S1368980011002102> |
| Heart disease | McClure, C. K., Catov, J. M., Ness, R. B., & Schwarz, E. B. (2012). Lactation and maternal subclinical cardiovascular disease among premenopausal women. *American Journal of Obstetrics and Gynecology*, *207*(1), 46.e1-46.e8. <https://doi.org/10.1016/j.ajog.2012.04.030>  Natland Fagerhaug, T., Forsmo, S., Jacobsen, G. W., Midthjell, K., Andersen, L. F., & Ivar Lund Nilsen, T. (2013). A prospective population-based cohort study of lactation and cardiovascular disease mortality: The HUNT study. *BMC Public Health*, *13*(1), 1070. <https://doi.org/10.1186/1471-2458-13-1070> |

**Table S2**

*Impact of Prenatal Breastfeeding Education on Breastfeeding Duration to 12+ Weeks: A Systematic Review*

| Database | Syntax |
| --- | --- |
| PubMed | ("Pregnancy"[Mesh] OR "Pregnant Women"[Mesh] OR "pregnan*"[Title/Abstract] OR "antenatal*"[Title/Abstract] OR "antepartum"[Title/Abstract] OR "prenatal*"[Title/Abstract]) AND ("Prenatal Education"[Mesh] OR "Prenatal Care"[Mesh] OR "Patient Education as Topic"[Mesh] OR "intervention*"[Title/Abstract] OR "support*"[Title/Abstract] OR "educat*"[Title/Abstract] OR "teach*"[Title/Abstract] OR "taught"[Title/Abstract]) AND ("Breast Feeding"[Mesh] OR "breastfe*"[Title/Abstract] OR "breast fe*"[Title/Abstract] OR "infant feed*"[Title/Abstract]) AND ("Time"[Mesh] OR "time"[Title/Abstract] OR "duration"[Title/Abstract]) AND ("random*"[Title/Abstract] OR "*experiment*"[Title/Abstract] OR "trial*"[Title/Abstract]) AND (2008/1/1:3000/12/12[pdat]) |
| MEDLINE | 1. exp Pregnancy/ or exp Pregnant Women/  2. ("pregnan*" or "antenatal*" or "antepartum" or "prenatal*").tw.  3. 1 or 2  4. Patient Education as Topic/ or Prenatal Care/ or Prenatal Education/  5. ("intervention*" or "support*" or "educat*" or "teach*" or "taught").tw.  6. 4 or 5  7. exp Breast Feeding/  8. ("breastfe*" or "breast fe*" or "infant feed*").tw.  9. 7 or 8  10. exp time/  11. ("time" or "duration").tw.  12. 10 or 11  13. 3 and 6 and 9 and 12  14. ("trial" or "*experiment*" or "random*").tw.  15. 13 and 14  16. limit 15 to yr="2008 -Current" |
| Embase | ('pregnancy'/exp OR 'pregnant woman'/exp OR 'pregnan*':ti,ab OR 'antenatal*':ti,ab OR 'antepartum':ti,ab OR 'prenatal*':ti,ab) AND ('health education'/exp OR 'intervention*':ti,ab OR 'support*':ti,ab OR 'educat*':ti,ab OR 'teach*':ti,ab OR 'taught':ti,ab) AND ('breast feeding'/exp OR 'breastfe*':ti,ab OR 'breast fe*':ti,ab OR 'infant feed*':ti,ab) AND ('time'/exp OR 'time':ti,ab OR 'duration':ti,ab) AND ('trial':ti,ab OR 'experiment*':ti,ab OR 'quasiexperiment*':ti,ab OR 'random*':ti,ab) AND (2008:py OR 2009:py OR 2010:py OR 2011:py OR 2012:py OR 2013:py OR 2014:py OR 2015:py OR 2016:py OR 2017:py OR 2018:py OR 2019:py OR 2020:py OR 2021:py OR 2022:py OR 2023:py) |
| CINAHL | S7 S1 AND S2 AND S3 AND S4 AND S5 Limiters - Published Date: 20080101-20231231  S6 S1 AND S2 AND S3 AND S4 AND S5  S5 TI ( ("trial" or "*experiment*" or "random*") ) OR AB ( ("trial" or "*experiment*" or "random*") )  S4 (MH "Time+") OR TI ( ("time" or "duration") ) OR AB ( ("time" or "duration") )  S3 (MH "Breast Feeding+") OR TI ( ("breastfe*" or "breast fe*" or "infant feed*") ) OR AB ( ("breastfe*" or "breast fe*" or "infant feed*") )  S2 ( (MH "Health Education+") or (MH "Prenatal Care+") or ) OR TI ( ("intervention*" or "support*" or "educat*" or "teach*" or "taught") ) OR AB ( ("intervention*" or "support*" or "educat*" or "teach*" or "taught") )  S1 ( (MH "Pregnancy+") or (MH "Expectant Parents+") ) OR TI ( ("pregnan*" or "antenatal*" or "antepartum" or "prenatal*") ) OR AB ( ("pregnan*" or "antenatal*" or "antepartum" or "prenatal*") ) |
| Cochrane | #1 MeSH descriptor: [Pregnancy] explode all trees  #2 MeSH descriptor: [Pregnant Women] explode all trees  #3 ("pregnan*" or "antenatal*" or "antepartum" or "prenatal*"):ti,ab,kw  #4 #1 or #2 or #3  #5 MeSH descriptor: [Patient Education as Topic] explode all trees  #6 MeSH descriptor: [Prenatal Care] explode all trees  #7 MeSH descriptor: [Prenatal Education] explode all trees  #8 ("intervention*" or "support*" or "educat*" or "teach*" or "taught"):ti,ab,kw  #9 #5 or #6 or #7 or #8  #10 MeSH descriptor: [Breast Feeding] explode all trees  #11 ("breastfe*" or "breast fe*" or "infant feed*"):ti,ab,kw  #12 #10 or #11  #13 MeSH descriptor: [Time] explode all trees  #14 ("time" or "duration"):ti,ab,kw  #15 #13 or #14  #16 ("trial" or "*experiment*" or "random*"):ti,ab,kw  #17 #4 and #9 and #12 and #15 and #16 |

**Table S3**

*Study Measures and Findings*

| **Study** | **Sample and Setting Details** | **Variables and Measures** | **Main Findings** | **Strengths, Weaknesses, and Quality Assessment Score** |
| --- | --- | --- | --- | --- |
| Antoñanzas-Baztán et al., 2021 | **Setting:** Two hospitals and two community clinics with pregnancy-related services in Spain  **Sample:** 123 pregnant women (63 intervention, 60 control)  48% primiparous, >70% married, >80% employed  Eligible if healthy woman in second trimester, attending regular check-ups, 18 yo+, intending to BFD after giving birth, with no BFG contraindications | **Independent:** Group assignment  *Intervention:* Given written information and viewed BSE-enhancing video during weeks 28-39 of pregnancy; during hospitalization after birth, given specific advice on items with a low score on BSES-SF and observation of whole BFG; within 48-72 h after discharge, follow-up call with verbal advice on low BSES-SF item  *Usual care control:* routine antenatal and PP visits  **Dependent:** Self-reported BFG status at 6 mos PP | BFG maintenance statistically significant in IG compared to CG at 6 mos PP (67% vs. 55%, X^2^  = 5.4, *p* = .02)  Log-rank test: significant difference in BFG survival between groups (X^2^ = 4.94, p = 0.026)  Participants with previous BFG experience of <6 mos had 8 times higher risk of giving up than those >6 mos (HR: 8.21 [1.65, 40.81], aHR: 7.8 [1.57, 38.7])  CG had about 3 times the risk of not continuing to BFD at 6-mo follow-up compared to IG | **Strengths:** Intent-to-treat analyses, followed participants to 6 mos  **Weaknesses:** Lack of randomization, significant loss to follow-up at 6-mo data collection point (30% intervention, 42% control), relatively small sample size  **Quality:** JBI Quasi-Experimental Checklist score = 8 |
| Bonuck et al., 2014 | **Setting:** Urban prenatal care sites in the Bronx, NYC, United States  **Sample:**  BINGO: 628 primarily low-income pregnant women (238 LC+EP, 236 EP, 77 LC, 77 usual care)    PAIRINGS: 262 economically-diverse pregnant women (129 LC+EP, 133 usual care)  Eligible if English- or Spanish-speaking, 18 yo+, in 1^st^ or 2^nd^ trimester with singleton, no risk factors for premature birth or maternal or infant conditions that would preclude/complicate BFG (HIV+, congenital anomaly) | **Independent:** Group assignment (BINGO: LC+EP, EP, LC, or usual care; PAIRINGS: LC+EP or usual care)  *Intervention EP:* electronic prompts appear in EMR during 5 prenatal visits  *Intervention LC:* 2 prenatal sessions, a hospital visit, regular phone calls PP through 3 mos or until BFG ceased, PP home visits optional  *Intervention LC+EP:* both EP and LC interventions  *Usual care:* access to routine hospital IBCLC  **Dependent:** Self-reported BFG at 3 and 6 mos | BINGO: any BFG differed by group at 3 mos and highest for the LC+EP and LC groups. LC+EP group had greater odds of 3 mos any BFG (OR = 2.10; 95% CI = 1.23, 3.61). EP group did not differ from CG on any outcome. No interaction between LC and EP for any BFG at 3 or 6 mos → no independent effect of EP on BFG outcomes. LC at 6 mos (OR = 1.84, CI [0.92, 3.68] *p* = .08)  PAIRINGS: IG had higher rates of any BFG at 3 (OR = 1.93, CI [1.17, 3.19] *p* = .01) and 6 mos (OR = 1.77, CI [1.03, 3.07] *p* = .04) | **Strengths:** 95% participant retention, ongoing visits scheduled into routing care rather than support only when sought by participant  **Weaknesses:** Sample not necessarily representative of US population of childbearing-age women; social desirability may bias participant reporting of BFG  **Quality:** van Tulder score = 8 |
| Cauble et al., 2021 | **Setting:** Kansas City-area OB/GYN practice; United States  **Sample:** 45 pregnant women (22 intervention, 23 control)  Eligible if primigravida or had exclusively BFD <3 mos; 9-30 wks GA; Excluded if fertility assistance, high risk for pre-term delivery, multiples, or morbid obesity (BMI > 40), diabetes (type 1, 2, or GDM), hypertension, metabolic dysfunction | **Independent:** Group assignment  *Intervention:* six 60-min sessions of prenatal group phone counseling with 6-10 participants led by IBCLC, assigned tasks for subsequent week  *Usual care:* standard pregnancy and pediatric education by HCP  **Dependent:** self-reported any BFG at 2 wks, 2, 4, and 6 mos | No significant difference found for rates of any BFG at any time point either per protocol or intention-to-treat  Intention-to-treat:  4 mos (*p* = .74)  6 mos (*p* = 1.0)  Per protocol:  4 mos (*p* = .56)  6 mos (*p* = .88) | **Strengths:** High response rate, high rate of compliance to protocol  **Weaknesses:** Homogeneity of sample and numerous exclusion criteria limit generalizability; small sample with lack of power; compliance defined as attending 4 of 6 meetings  **Quality:** van Tulder score = 8 |
| Chan et al., 2016 | **Setting:** Obstetric unit of university-affiliated public hospital in Hong Kong  **Sample:** 71 pregnant women (35 intervention, 36 control)  100% married  Eligible if primiparous, 18 yo+, married, normal breast/nipple exam results, no anticipated medical or pregnancy complications that inhibit BFG, able to understand and communicate in Chinese | **Independent:** Group assignment  *Intervention:* 2.5-hr interactive BFG workshop at 28-38 wks GA, groups of 6-8; telephone counseling at 2 wks PP, each call 30–60 mins  *Usual care:* BFG support from midwives in the hospital, PP follow-up  **Dependent:** Self-reported BFG duration at 3, 4, and 8 wks and 6 mos PP | Log-rank test: no significant difference in BFG survival time between the two groups (χ2=2.03, p=0.07), although 31.4% of the mothers in IG BFD their babies at 6 mos PP compared with 16.7% of the mothers in CG. | **Strengths:** Intent-to-treat survival analyses of BFG duration  **Weaknesses:** Small sample size, not powered to detect difference despite almost double the percent of mothers BFG at 6 mos in IG compared to CG, high refusal rate (39%), participating mothers may have already been highly motivated to BFD  **Quality:** van Tulder score = 8 |
| Efrat et al., 2015 | **Setting:** 5 community health clinics in Los Angeles County, United States  **Sample:** 289 pregnant women (146 intervention, 143 control)  54% married, <20% some college+  Eligible if 26-34 wks GA, Medicaid recipient, Hispanic, telephone available, not assigned to WIC peer counselor | **Independent:** Group assignment  *Intervention:* 4 prenatal and 17 PP phone calls (most 5-7 mins); 2 prenatal contacts focused on equipping with critical BFG knowledge  *Usual care:* routine BFG education and support offered by health system  **Dependent:** self-reported BFG endpoint (the last date baby at breast), contacted at 3 days, 1, 3, and 6 mos PP | There were no significant differences between groups in the any BFG category at any time points. Mean duration 20 wks ± 9.2 for CG vs. 20.7 wks ± 8.9 for IG (*p* = .716)  3 mos any BFG 94.6% CG vs. 98.2% IG (*p* = .25)  6 mos any BFG 85.8% CG vs. 94.4% IG (*p* = .17) | **Strengths:** more feasible and cost-conscious than many other interventions  **Weaknesses:** CIs not given for ORs, confusing data presentation, disparate intentions to BFD between groups at baseline, underpowered, not blinded – research assistants were also the lactation educators  **Quality:** van Tulder score = 4 |
| Jiang et al., 2014 | **Setting:** 4 community health centers in Shanghai, China  **Sample:** 582 pregnant women (281 intervention, 301 control)  Eligible if first trimester, owned mobile phone, first-time mother, singleton fetus, older than 20 y, completed at least compulsory jr. high education, no illness that limited BFG after childbirth | **Independent:** Group assignment  *Intervention:* Once weekly text message with relevant BFG or infant feeding advice from 28 wks GA to 12 mos PP  *Usual care:* usual health care services  **Dependent:** self-reported BFG duration at 4, 6, and 12 mos PP | Median duration of any BFG at 12 mos almost identical: 7.72 mos IG (95% CI, 7.26-8.19) vs. 7.73 mos CG (95% CI, 7.28-8.18) *p* = .94, log-rank test. Cox proportional hazards analysis detected no difference in stopping BFG between IG and CG (HR, 1.00 [95% CI, 0.82-1.19]) | **Strengths:** intervention is likely to be cost-effective and relatively easy to implement  **Weaknesses:** quasi-experimental design with group differences, high education level of participants limits generalizability  **Quality:** JBI Quasi-Experimental Checklist score = 7 |
| Kellams et al., 2018 | **Setting:** University of Virginia and Virginia Commonwealth University Health Systems, United States  **Sample:** 522 low-income, pregnant women (263 intervention, 259 control);  Excluded if multiple gestation, known contraindication to BFG, or unable to speak English | **Independent:** Group assignment  *Intervention:* 25-min educational BFG video viewed at prenatal visit  *Control:* 20-min educational nutrition/exercise video  **Dependent:** any BFG during birth hospitalization from hospital records; self-reported any BFG at 1, 3, and 6 mos | Duration of BFG did not differ by IG over 1st 6 mos; women in the IG and CG stopped BFG at same rate; when stratified by previous BFG experience, there were no significant differences in duration  HR = 1.00 (CI: 0.81–1.24)  aHR = 0.99 (CI: 0.79–1.24) | **Strengths:** large sample, low-cost and easy-to-implement intervention  **Weaknesses:** limited generalizability (all low-income); non-English speaking women excluded  **Quality:** van Tulder score = 10 |
| Kronborg et al., 2012 | **Setting:** Large midwifery clinic of university hospital in an urban area of Denmark  **Sample:** 1193 pregnant women (590 control, 603 intervention)  Most women live with partners  Eligible if first pregnancy, older than 18 y, singleton pregnancy, able to speak and understand Danish | **Independent:** Group assignment  *Intervention:* Ready for Child Programme; 3 modules, 3 hrs each at 30-35 wks GA, partner invited, up to 8 couples per class, lecture and discussions; BFG part ~2 hrs  *Usual care:* standard care offered by the antenatal clinic  **Dependent:** self-reported BFG duration in weeks assessed at 1yr PP | Found no difference in the two groups according to the women’s reports of duration of any breast feeding assessed at 12 months (HR = 0.96 CI: 0.84–1.09). | **Strengths:** large sample size, intent-to-treat analysis, RCT  **Weaknesses:** many in CG were independently seeking out antenatal training, population and sample quite homogenous (well-educated, white, area of high BFG rates)  **Quality:** van Tulder score = 7 |
| Meedya et al., 2014 | **Setting:** Tertiary, metropolitan hospital in Sydney, Australia  **Sample:** 366 pregnant women (172 intervention, 194 control)  About half born outside Australia, 85.5% in a committed relationship (married or de-facto), 83.6% intended to breast feed for about 6 mos or more  Eligible if nulliparous, 19 yo+, planned to BFD, basic English literacy | **Independent:** Group assignment  *Intervention:* Milky Way program elements: group sessions, take-home learning activities, and postnatal telephone consultations to support BFG (one within the first 10 days and the other about 3 mos PP). Group sessions started in the second trimester. Women and partner/support person participated in groups of 10–20 people.  *Usual care:* Standard maternity care  **Dependent:** BFG status at 4 and 6 mos | At 6 mos, IG group had higher rates of BFG compared with the CG, 54.3% vs 31.4%, p<0.001  At 4 mos, 64.5%, IG vs. 37.1% CG, p<0.001.  Effect sizes (calculated without missing data): 6 mos moderate (Cramer’s Phi =0.38); 4 mos moderate (Cramer’s Phi =0.31).  Intervention increased likelihood of any BFG by 4x at 4 mos (OR= 4.13 [2.48–6.89]) and 3x at 6 mos (OR=3.01 [1.86–4.86]) | **Strengths:** intent-to-treat analyses, large sample for intensity of intervention  **Weaknesses:** lack of blinding, limited generalizability, high intensity intervention may not be cost-effective  **Quality:** JBI Quasi-Experimental Checklist score = 8 |
| Mikami et al., 2017 | **Setting:** Multiple pregnancy unit in Sao Paulo, Brazil  **Sample:** 171 women pregnant with twins (88 intervention, 83 control)  About 40% nulliparous  Eligible if both fetuses alive at 18-34 wks GA and followed at twin clinic, no lactation inhibitors to treat prolactinoma, and no major malformations that could preclude BFG | **Independent:** Group assignment  *Intervention:* 3 30-min small group BFG-specific counseling sessions of 2-3 women provided by 1 of 2 midwives  *Usual care:* routine antenatal care protocol for twin pregnancies  **Dependent:** Self-reported any BFG at 90 days and 180 days PP | No significant differences in rates of any BFG between groups at any time period.  90 days: OR = 1.50 [0.72, 3.10]  180 days: OR = 1.06 [0.51, 2.19] | **Strengths:** Unique population (mothers of twins)  **Weaknesses:** Sample size too small to detect less than 25% increase in BFG rates; 25% lost to follow-up and excluded from analyses, unknown compliance  **Quality:** van Tulder score = 4 |
| Nabulsi et al., 2019 | **Setting:** Two academic tertiary care centers in Beirut, Lebanon  **Sample:** 362 pregnant women (174 intervention, 188 control)  About half employed  Eligible if healthy pregnancy in 1st or 2nd trimester, intention to attempt to BFD;  Excluded if maternal chronic condition, abnormal fetal screen, multiples, delivery before 37 wks | **Independent:** Group assignment  *Intervention:* intervention components: a) prenatal BFG education, b) PP professional lactation support, c) PP peer (lay) support  *Usual care:* standard prenatal and postnatal care provided OBs only  **Dependent:** self-reported any BFG at 3 and 6 mos | No significant difference in crude BFG rates at 3 mos (78.7% CG vs. 76.6% IG, *p* = .64) or 6 mos (61.6% CG vs. 59.1% IG, *p* = .65)  Multivariable logistic regression adjusted for variables like site, income, rooming in, BFG behavior, duration of previous BFG, number of children, number children BFD, and group allocation, participants in IG twice as likely to continue EBF for 6 mos but similar model not used to evaluate any BFG | **Strengths:** relatively large sample, multi-component intervention  **Weaknesses:** only 39/174 completed all 3 intervention components, all participants from urban setting, participant blinding not fully possible as those with previous children could guess group assignment, lack of outcome assessor blinding  **Quality:** van Tulder score = 6 |
| Puharić et al., 2020 | **Setting:** 8 obstetric practices, half private and half public, in Split-Dalmatia County, Croatia  **Sample:** 400 nulliparous pregnant women (136 intervention, 128 active control, 136 standard care)  99% planned to feed exclusively or partially breast milk, 64% planned to breastfeed > 6 mos  Eligible if primigravida, singleton, attended primary care obstetrician 20-32 wks GA, able to speak Croatian, reside within Republic of Croatia for at least 1 yr | **Independent:** Group assignment  *Intervention:* received BFG booklet and general pregnancy booklet, followed by 4 proactive phone calls –1 in pregnancy and 3 PP at 2, 6, and 10 wks  *Active control:* received general pregnancy booklet, followed by 4 proactive phone calls – 1 in pregnancy and 3 PP at 2, 6 and 10 wks  *Usual care:* received standard care  **Dependent:** self-reported BFG duration by postal survey at 3 and 6 mos PP | For participants in the usual care group, the odds of not BFG at 3 mos were 4.6x that of those in the IG (OR = 4.6 [2.7, 8.1])  For participants in the usual care group, the odds of not BFG at 6 mos are 15.7x that of those in the IG (OR = 15.7 [9.1, 27.1]) | **Strengths:** plain language written materials that were pilot tested by 40 pregnant women, comparable groups at baseline, minimal attrition  **Weaknesses:** ordinal regression rather than Kaplan-Meier survival or Cox proportional hazards analyses  **Quality:** van Tulder score = 8 |
| Schreck et al., 2017 | **Setting:** Urban teaching hospital in Detroit, MI, United States  **Sample:** 650 pregnant women (320 intervention, 330 control)  87% Black, higher NICU admission rate in controls (16.1 vs 7.3%), more in CG had previous BFG experience (47.9 vs 37.6%); did not have to intend to BFD to enroll | **Independent:** Group assignment  *Intervention:* BFG-focused prenatal education curriculum of up to 10 visits at site-associated resident prenatal clinic delivered one-on-one by an IBCLC; subgroup optionally attended BFG support group  *Usual care:* care in prenatal clinic before intervention implemented  **Dependent:** Self-reported BFG duration and status at 6 mos | BFG continuation ≥ 6 mos was not affected by prenatal education only; T test: mean CG duration = 4.3 mos ± 4.9 vs mean IG duration = 4.0 mos ± 4.2, *p* = 0.5  Participation in both prenatal education and peer support group increased BFG duration. 59% IG who participated in both breastfed for at least 6 mos vs. 28% IG with prenatal education alone (*p* = .002) | **Strengths:** Large sample, prolonged follow-up; most in IG reported prenatal BFG education had impact on feeding decisions  **Weaknesses:** No clear inclusion/exclusion criteria; 10 visits offered but mean was 3.2 visits; no statistical tests control for variables that differ between groups  **Quality:** JBI Quasi-Experimental Checklist score = 8 |
| Stuebe et al., 2016 | **Setting:** 2 Raleigh, NC (United States) clinics, 1 primarily privately-insured clients, the other primarily publicly insured clients  **Sample:** 100 pregnant women (50 intervention, 50 control)  Eligible if diagnosed with GDM, at least 22 but less than 37 wks GA, age ≥18 and ≤45, pre-pregnancy BMI >25 kg/m^2^, able to read/write in English; excluded if overt diabetes (baseline A1c ≥ 6.5 mg/dL) | **Independent:** Group assignment  *Intervention:* NEST group intervention beginning with a prenatal BFG class. Then, starting ~6 wks PP, women participated in a 13-wk intensive lifestyle intervention delivered with weekly classes and a home exercise program.  *Waitlist control:* usual care for lactation support and GDM  **Dependent:** self-reported BFG duration in days at 4, 7, and 10 mos PP | Women in the IG were more likely to be BFG (log rank p < 0.01) throughout follow-up period.  In proportional hazards modeling, IG less likely to discontinue BFG (unadjusted HR 0.44 [0.21, 0.95]. Association strengthened with adjustment for prenatal infant feeding intentions, site, and Black race → aHR = 0.40 [0.21–0.74]  Censored for the Kaplan–Meier analyses and Cox modeling if lost to follow-up before discontinuing any or exclusive BFG or if still BFG at study end | **Strengths:** Intent-to-treat analyses, theory-based intervention  **Weaknesses:** High loss to follow-up, significant difference in race between groups despite randomization (more Black participants in CG and more White participants in IG, *p* = .02)  **Quality:** van Tulder score = 5 |
| Tseng et al., 2020 | **Setting:** Prenatal clinic of teaching hospital in Taiwan  **Sample:** 104 pregnant women (52 intervention, 52 control)  Most highly educated, married, & employed full-time  Eligible if ≥ 20 yo, primipara, singleton at 12–32 wks GA, willing to BFD, and husband/partner willing to attend program; Excluded if had chronic disease, pre-term labor, abnormal fetal screening, or other high-risk condition | **Independent:** Group assignment  *Intervention:* integrated BFG education program based on theory of self-efficacy, 3 2.5-hr sessions of 4-5 couples at 34, 35, and 36 wks GA, simulation and mindfulness components  *Usual care:* standard usual care provided at study site hospital based on Baby Friendly Hospital Initiative  **Dependent:** Self-reported exclusive + predominant BFG vs bottle feeding at 1 wk, 1, 3, and 6 mos | At 3 mos PP, IG exhibited significantly higher rates of exclusive or predominant BFG compared to CG  Chi-square:  3 mos (*p* = .02)  6 mos (*p* = .07) | **Strengths:** Simulation and mindfulness are novel components  **Weaknesses:** Bottle feeding group may include those feeding breast milk by bottle; small sample size with significant loss to follow-up in CG  **Quality:** van Tulder score = 8 |
| van Dellen et al., 2019 | **Setting:** The Netherlands  **Sample:** 138 pregnant women (66 intervention, 72 control)  Eligible if pregnant, planning to BFD, has access to internet, singleton gestation, non-missing data for BFG duration | **Independent:** Group assignment  *Intervention:* Series of 6 consults up to 10 wks PP, mix of in-person and phone calls  *Usual care:* Usual obstetric and PP care  **Dependent:** self-reported BFG status assessed at 28 wks post-due date | Any BFG survival rates significantly higher in IG group than in CG (log-rank test: χ2(1) = 4.79, p = .029)  Effect of intervention on survival rates for any BFG controlling for baseline differences (HR = 0.34, [0.18–0.61]).  Nulliparous subgroup: Any BFG survival rates higher in IG than CG (log-rank test: χ2(1) = 7.85, p = .005) Mean BFG duration 27.5 wks in IG vs 19.5 wks CG; Controlling for variables which differed at baseline, effect of intervention on survival rates for any BFG in same direction but no longer significant (HR = 0.42, p = .113 [0.15,1.23]) | **Strengths:** Comprehensive BFG interventions combining support and education, incorporates evidence-based elements  **Weaknesses:** lack of randomization, unclear generalizability, minimal info given on setting of study and recruitment sites  **Quality:** JBI Quasi-Experimental Checklist score = 7 |
| Wambach et al., 2011 | **Setting:** Kansas City (United States) prenatal clinics, high schools, and hospitals  **Sample:** 390 pregnant teens (128 intervention, 128 attention control, 134 usual care)  only followed BFG participants after hospital discharge (201 total with 77 intervention, 60 attention control, 64 usual care), 61% Black, 74% single/living with family, 71% in school  Eligible if 2nd trimester, 15-18 yo, primigravida, planning to keep newborn | **Independent:** Group assignment  *Intervention:* prenatal, in-hospital, & PP education & support. 2 prenatal classes (1 1.5 h, 1 2 hr), PC calls before and after class 1 and after class 2. Visit from PC in hospital after birth. BFG teens visited by IBCLC. PP phone contact at 4, 7, 11, 18 days and 4 wks  *Attention control:* paralleled the IG in content amount/timing; no BFG focus  *Usual care:* received standard prenatal and PP care at their respective clinic  **Dependent:** self-reported BFG duration in days | Median BFG duration was 177, 42, and 61 days for IG, the attention control, and the usual care groups. Log-rank test: BFG duration longer among those in IG compared to either CG, χ2(df = 2) = 16.26, p < .001.  IG effect remained significant (p = .015) after adjusting for positive BFG sentiment and social and professional support using the Cox Proportional hazards. | **Strengths:** intention-to-treat duration analysis, teen-focused intervention  **Weaknesses:** significant participant attrition, possible recall bias. Dropped from study if did not attend at least one class  **Quality:** van Tulder score = 5 |
| Wen et al., 2011 | **Setting:** Socially and economically disadvantaged areas of Southwest Sydney, Australia, antenatal clinics of Liverpool and Campbelltown hospitals  **Sample:** 667 nulliparous pregnant women (330 control, 337 intervention)  87.3% either married or living with partner, 20.7% unemployed  Eligible if 16 yo+, 24-34 wks GA. Excluded if severe medical condition. | **Independent:** Group assignment  *Intervention:* 1 home visit at 30-36 wks GA and 5 home visits at 1, 3, 5, 9, and 12 mos PP. At each visit, research nurse spent 1-2 hrs with the dyad.  *Usual care:* usual childhood nursing service, 1 home visit within a month of birth if needed  **Dependent:** self-reported BFG duration at 6 and 12 mos | BFG rates higher in IG than CG at 6 and 12 mos (42.2% vs 32.1% and 21.0% vs 14.9%); Median BFG duration 17 wks IG [13.9, 20.4] vs. 13 wks CG [10.1, 15.6 weeks] (*p*=.03, log-rank test) HR for stopping BFG in intervention HR = 0.82 [0.68, 0.99]  Subgroup (antenatal intervention): effect on BFG significant (*p*=.009) and appeared early (around 2 wks), with a 25% reduced risk of stopping BFG in IG (HR = 0.75 [0.60, 0.93]). If without antenatal intervention, effect appeared later, at ~12 wks, and was not significant during the entire year (HR =0.96 [0.74, 1.24]) | **Strengths:** Intervention designed to ensure consistency of health information delivery, relatively large sample size  **Weaknesses:** Not all participants received antenatal portion of intervention (only 35.3% received the antenatal visit); those lost to follow-up were significantly younger and less educated and were more likely to be unemployed or have low income; cost and time-intensive intervention  **Quality:** van Tulder score = 7 |
| Wen et al., 2020 | **Setting:** Metropolitan Sydney, New South Wales, Australia, antenatal clinics in 7 hospitals of 4 local health districts  **Sample:** 1155 pregnant women (385 control, 386 telephone intervention, 384 SMS intervention)  55% speak English at home, 90+% married, 54% first-time mothers  Eligible if 16y+, 24-24 wks GA, able to communicate in English, access to mobile telephone | **Independent:** Group assignment  *Both IGs:* 1 intervention in 3rd trimester and 5 interventions at 1, 3, 5, 7, 10 mos PP. Staged booklets mailed to match timing of support  *Telephone intervention:* 1-wk post-booklet call (30-60 min) for support and to discuss booklet and any issues  *SMS intervention:* 1wk after booklet, messages sent 2x/wk for 4 wks via 2-way SMS to reinforce key messages  *Usual care:* usual care from local health nurses, home safety materials sent to CG in 3^rd^ trimester, 3, 6, and 9 mos PP  **Dependent**: self-reported BFG duration asked at 6 mos and 12 mos | Rates of BFG at 6mos were similar between the groups (70% phone, 71% SMS, 68% CG; aOR phone vs control 1.14 [0.80,1.64], aOR SMS vs control 1.08 [0.91, 1.27])  Rates of BFG at 12 months were higher in the phone support group (49%) and the SMS support group (49%) than in the CG (44%), but not statistically significant, aOR phone vs control 1.25 [0.91,1.72], aOR SMS vs control 1.11 [0.95, 1.30] | **Strengths:** large sample size, published study protocol  **Weaknesses:** unable to address many social, cultural, economic, and environmental factors associated with infant feeding practices, 30% of telephone intervention participants did not receive all of the phone support sessions; high withdrawal rate from telephone IG  **Quality:** van Tulder score = 8 |
| Wong et al., 2014 | **Setting:** Antenatal clinics of two geographically distributed public hospitals in Hong Kong  **Sample:** 469 pregnant women (233 intervention, 236 control)  Eligible if 18 yo+, Cantonese-speaking, nulliparous, 35 wk+ GA, singleton pregnancy, no serious medical/obstetric complications, intent to BFD, in Hong Kong for at least 6 mos after birth | **Independent:** Group assignment  *Intervention:* 20-30 min 1-to-1 BFG education and support session based on World Health Organization guidelines; delivered immediately after randomization in a private room in the antenatal clinics, handouts given  *Usual care:* standard antenatal care  **Dependent:** self-reported duration in weeks at 6 wks, 3 mos, and 6 mos PP | Any BFG rates at 6 wks (68.7% vs 71.6% [20.13, 0.06]), 3 mos (49.8% vs 55.5% [20.15, 0.03)), 6 mos (37.3% vs 40.7% [20.13, 0.06]) not significantly different between IG and CG. No significant differences between IG and CG in overall duration of any (HR 1.11, 95% CI 0.88–1.40) BFG across 6-mo follow-up period. | **Strengths:** simple intervention delivered at time of enrollment  **Weaknesses:** limited generalizability  **Quality:** van Tulder score = 8 |
| Zhao et al., 2021 | **Setting:** Urban hospital in Shanghai, China  **Sample:** 182 pregnant women (91 intervention, 91 control)  Must have EPDS score ≥9 and be married  Excluded if met criteria for major depression disorder | **Independent:** Group assignment  *Intervention:* individualized mixed management psychoeducational intervention focused on perinatal mental health and BFG, delivered in four 60-min face-to-face sessions  *Usual care:* routine obstetric examination and follow-up  **Dependent:** Self-reported feeding pattern at 3 mos and 6 mos PP | Feeding patterns were significantly different at all time periods with more BFG in IG. When comparing any BFG to no BFG, significant differences remained only at 42 days and 6 mos.  Chi-square, 5 feeding categories:  3 mos (*p* = .033)  6 mos (*p* = .022)  Chi-square, any BFG vs no BFG:  3 mos (*p* = .443)  6 mos (*p* = .021) | **Strengths:** assessed group equivalence across numerous variables; 1-to-1 individualized intervention; 100% compliance reported  **Weaknesses:** older, educated sample; highly specific inclusion criteria – only primigravidas, required to be married, required to have EPDS ≥9 but without meeting criteria for major depression disorder; resource intensive intervention; intervention not exclusively BFG content  **Quality:** van Tulder score = 6 |

*Note.* aHR = adjusted hazard ration; aOR = adjusted odds ratio; BFD = breastfeed/breastfed; BFG = breastfeeding; BINGO = best infant nutrition for good outcomes; BMI = body mass index; BSE = breastfeeding self-efficacy; BSES-SF = Breastfeeding Self-Efficacy Scale – Short Form; CG = control group; CI = confidence interval; EMR = electronic medical record; EP = electronic prompts; EPDS = Edinburgh Postnatal Depression Scale; GA = gestational age; GDM = gestational diabetes mellitus; GYN = gynecology; HCP = healthcare provider; HR = hazard ratio; IBCLC = International Board Certified Lactation Consultant; IG = intervention group; JBI = Joanna Briggs Institute; LC = lactation consultant; NEST = nutrition, exercise, and social cognitive theory-based; NICU = neonatal intensive care unit; OB = obstetrician/obstetrics; OR = odds ratio; PAIRINGS = Provider Approaches to Improved Rates of Infant Nutrition and Growth Study; PC = peer counselor; PP = postpartum; RCT = randomized controlled trial; SMS = short message service; WIC = Special Supplemental Nutrition Program for Women, Infants, and Children.
